# The essential role of transcription factor Pitx3 in preventing mesodiencephalic dopaminergic neurodegeneration and maintaining neuronal subtype identities during aging

**DOI:** 10.1038/s41419-021-04319-x

**Published:** 2021-10-27

**Authors:** Ying Wang, Xi Chen, Yuanyuan Wang, Song Li, Huaibin Cai, Weidong Le

**Affiliations:** 1 Liaoning Provincial Key Laboratory for Research on the Pathogenic Mechanisms of Neurological Diseases, the First Affiliated Hospital, Dalian Medical University, Dalian, 116011 China; 2 Institute of Neurology and Department of Neurology, Sichuan Academy of Medical Sciences-Sichuan Provincial Hospital, Medical School of UETSC, Chengdu, 610072 China; 3 Transgenic Section, Laboratory of Neurogenetics, National Institute on Aging, National Institutes of Health, Bethesda, MD 20892 USA

**Keywords:** Neuroscience, Neurological disorders

## Abstract

*Pituitary homeobox 3* (*Pitx3*) is required for the terminal differentiation of nigrostriatal dopaminergic neurons during neuronal development. However, whether Pitx3 contributes to the normal physiological function and cell-type identity of adult neurons remains unknown. To explore the role of Pitx3 in maintaining mature neurons, we selectively deleted *Pitx3* in the mesodiencephalic dopaminergic (mdDA) neurons of *Pitx3*^*fl/fl/*^*DAT*^*CreERT2*^ bigenic mice using a tamoxifen inducible *Cre*^*ERT2*/*loxp*^ gene-targeting system. *Pitx3*^*fl/fl/*^*DAT*^*CreERT2*^ mice developed age-dependent progressive motor deficits, concomitant with a rapid reduction of striatal dopamine (DA) content and a profound loss of mdDA neurons in the substantia nigra pars compacta (SNc) but not in the adjacent ventral tegmental area (VTA), recapitulating the canonical neuropathological features of Parkinson’s disease (PD). Mechanistic studies showed that *Pitx3*-deficiency significantly increased the number of cleaved caspase-3^+^ cells in SNc, which likely underwent neurodegeneration. Meanwhile, the vulnerability of SNc mdDA neurons was increased in *Pitx3*^*fl/fl/*^*DAT*^*CreERT2*^ mice, as indicated by an early decline in glial cell line-derived neurotrophic factor (GDNF) and aldehyde dehydrogenase 1a1 (Aldh1a1) levels. Noticeably, somatic accumulation of α-synuclein (α-syn) was also significantly increased in the *Pitx3*-deficient neurons. Together, our data demonstrate that the loss of Pitx3 in fully differentiated mdDA neurons results in progressive neurodegeneration, indicating the importance of the *Pitx3* gene in adult neuronal survival. Our findings also suggest that distinct Pitx3-dependent pathways exist in SNc and VTA mdDA neurons, correlating with the differential vulnerability of SNc and VTA mdDA neurons in the absence of *Pitx3*.

## Introduction

The main pathological characteristic of PD is a profound loss of mdDA neurons in SNc [1, 2]. However, the proximal neurons within VTA and retrorubral field (RRF) are less vulnerable to degeneration and are largely spared during the course of the disease [3]. Therefore, some inherent and different gene expression profiles of mdDA neuron subtypes impact their distinct vulnerability to PD pathology, i.e., a molecular code may be rooted in the neuroepithelium to specify the subsets of mdDA neurons [4].

During neuronal development, mdDA precursors are thought to originate from the floor plate of the midbrain [5]. Temporal and spatial studies suggest that SNc neurons are derived from a rostral neuronal population earlier, whereas VTA neurons from a caudal one later [6]. A cascade of developmental transcription factors has long been known to define the mdDA neuronal fate specification in the neuroepithelium, including Pitx3, Nurr1, Engrailed, and Lmx1b [7, 8]. Particularly, these molecules are not only involved in the early neuronal events but are also continuously expressed during neuronal maturation and even throughout adulthood [9]. However, how these transcription factors play a role in modulating the postnatal neurons is still largely unknown, a stark contrast to their well-documented involvement in the developmental stages [3]. Conventional knockout mice of *Nurr1* die shortly after birth [10], whereas recent studies have shown that the haploinsufficient ones can survive until the late stages, given exhibiting mdDA neuron loss and locomotor deficits [11]. This *Nurr1*-related neuronal loss might be largely attributed to the intrinsic dysregulation of *Bcl-2* and *P53* genes, both critical for cell survival [12, 13]. Furthermore, the conditional deletion of *Nurr1* in mature neurons results in a rapid reduction of striatal DA, a loss of mdDA neuronal markers, and neuronal degeneration [14, 15].

Pitx3, another transcription factor, has also gained particular interest, since its polymorphisms were genetically linked to PD cases [16, 17]. During mouse development, Pitx3 is expressed in the eye lens, skeletal muscles, and mdDA neurons [18, 19]. However, after birth, its expression is preserved only in mdDA neurons [18, 19], indicating that Pitx3 plays an important role in both development and maintenance of these neurons. Moreover, Aphakia (*ak*) mice, which are *Pitx3*-deficient, display SNc mdDA neuron preferential loss during embryonic and postnatal development [20], recapitulating Pitx3 is highly involved in early mdDA neuronal events. However, compared with Nurr1, the role of Pitx3 in mature mdDA neurons has not been studied. As an initial attempt to understand the role of Pitx3 in maintaining mature mdDA neurons, we established a *Pitx3*^*fl/fl*^/*DAT*^*CreERT2*^ (*Pitx3*^*cKO*^) mouse model that postnatally knocks out the *Pitx3* gene in the cells expressing the dopamine transporter (DAT) protein under tamoxifen (TAM) treatment. Our results showed that the conditional deletion of *Pitx3* in adult mice caused striatal DA reduction and locomotor activity abnormalities. Moreover, a significant loss of SNc mdDA neurons was first noted at 9 months after TAM treatment in *Pitx3*^*cKO*^ mice and was further aggravated at 15 months of age accompanied by a significant increase of cleaved caspase 3^+^ cells in SNc. In contrast to *ak* mice [20, 21], our mouse model showed that *TH*, *DAT*, and *Nurr1*, the hallmarks of mdDA neurons were less affected by *Pitx3* within the adult VTA neurons during aging. However, *GDNF*, *BDNF*, and *Aldh1a1*, which are *Pitx3*-related genes, were all substantially reduced in both the SNc and VTA mdDA neurons at the early or/and advanced stages. Such distinct expression profiles of candidate genes within SNc and VTA may contribute to the differential vulnerabilities of neuronal subtypes to the loss of Pitx3. Interestingly, the somatic accumulation of α-syn was markedly increased in the mdDA neurons of 15-month-old *Pitx3*^*cKO*^ mice. These data altogether establish the key role of Pitx3 in maintaining the normal physiological functions and preserving the specific molecular identities of postnatal mdDA neurons.

## Methods

### The generation of conditional knockout *Pitx3* mouse model

The heterozygous mice *Pitx3*^Flox/wt^ with C57BL/6J background were generated by ViewSolid Biotech Co., Ltd. (Beijing, China). To achieve the conditional knockout *Pitx3* mouse model in the mdDA neuronal system, *Pitx3*^*fl/fl*^*/DAT*^*CreERT2*^ mice were produced by breeding mice carrying an inducible Cre recombinase under the *DAT* promoter with the heterozygous mice *Pitx3*^Flox/wt^. The *DAT*^*CreERT2*^ mice were kindly gifted by the Günther Schütz Group (German Cancer Research Center) [22], which were generated by recombining a construct containing an improved Cre recombinase fused to a modified ligand-binding domain of the estrogen receptor into a bacterial artificial chromosome containing the gene encoding DAT.

All experimental mice were maintained under specific-pathogen-free (SPF) conditions (temperature, 22°C ± 2°C; air exchange, per 20 min; 12 h/12 h light–dark cycle) with free access to food and water. Animal care and procedures were carried out in accordance with the Laboratory Animal Care Guidelines approved by the Institutional Animal Care Committee at Dalian Medical University. The protocol was approved by the Institutional Animal Care Committee at Dalian Medical University.

TAM (T-5648; Sigma-Aldrich) was dissolved in corn oil (S-5007, Sigma-Aldrich) and ethanol mixture with the ratio of 10:1. A fresh mixture was prepared by shaking overnight to dissolve TAM completely, and the mixture was then stored in a dark place at 4 °C. Inducible Cre recombinase was activated in 2-month-old transgenic mice by administering TAM as 2 mg/day via intraperitoneal injection for five consecutive days [23].

For the performance of genotyping, *DAT*^*CreERT2*^ mice were identified by a PCR assay (2XEasyTaq PCR SuperMix, AS-111, Transgen Biotech) via tail biopsy. The sequence of the forward primer is AGA ACC TGA TGG ACA TGT TCA GG and the sequence of the reverse primer is CAG ACC AGG CCA GGT ATC TCT. The length of the target amplicon is 700 bp. PCR program: 95 °C for 45 s; 40X (95 °C for 15 s, 60 °C for 20 s, 72 °C for 30 s); 72 °C for 1 min; 4 °C for 2 min; hold at 4 °C. *Pitx3*^*Flox/wt*^ mice were identified by a PCR assay (2XEasyTaq PCR SuperMix, AS-111, Transgen Biotech) via tail biopsy. The sequence of the forward primer is GTC AGT GGA TAG GAA AAG AGG C and the sequence of the reverse primer is TCA CTC TAC AGT GTG TAC CTG GTC. The PCR product size of wild-type allele is 167 bp and mutant allele is 201 bp. PCR program: 94 °C for 2 min; 32X (98 °C for 10 s, 60 °C for 30 s, 68 °C for 50 s); 68 °C for 5 min; hold at 16 °C.

### Behavioral test

The open-field test was performed in a quiet testing room over 3 continuous days. To measure the locomotor activity, mice were placed into an Activity Monitor instrument (25 × 25 × 30 cm, Med Associates Inc., St. Albans, USA) equipped with computer-controlled photocells. Locomotor activity was automatically recorded for 40 min, and the total distance traveled and the number of rearings were calculated by the Med system.

The pole test was performed as described previously [14]. Mice were placed head-up on top of a vertical pole and allowed to descend freely to the bottom of the box with 1-day training before the test. On the test, the animals underwent three trials, and the time to orient downward (t-turn) and the total time to turn and descend the pole to the floor (t-total) were measured.

Rotarod assays were performed using the rotarod apparatus (Model 755, IITC Life Science). Both training and testing were performed at three trials per day for 3 consecutive days, as described previously [20]. At the start of the test, mice were stood on the rod and allowed to habituate for 1 min. After that, mice were trained to attain stable baseline levels of performance and climbing on the rod rotating at a constant speed of 4 rpm for 5 min. Subsequently, a protocol with an acceleration of 6 rpm/min was applied at a maximum speed of 40 rpm. The duration time for each mouse was then recorded.

### Immunostaining

Mouse brains were collected at indicated time points after TAM injections. Mice were anesthetized by isoflurane with chloral hydrate perfused through the left ventricle with PBS. The brains were rapidly isolated and postfixed in ice-cold 4% paraformaldehyde and subsequently dehydrated for 24 h in 15% and 30% sucrose at 4 °C, as described previously [24].

For immunohistochemical (IHC) staining, rabbit (PV-9001, ZSGB-BIO Company, Beijing, China) or mouse (PV-9002, ZSGB-BIO Company, Beijing, China) two-step detection kit was used, as described previously [25]. A series of slides were incubated in Solution A for 10 min. After rinsing with PBS three times, blocking solution (5% normal goat serum, 0.2% Triton-X 100, and 0.05% NaN_3_ in PBS) was applied for 1 h at room temperature. The primary antibodies were used as follows: anti-TH (1:1000, AB152; Millipore, USA) and anti-α-syn (1:1000, 610786; BD Transduction Laboratories, USA). The use of Solutions B and C were performed according to manufacturer’s instructions and after subsequent exposure to diaminobenzidine (ZLI-9019, ZSGB-BIO Company, China) for 5 min. After rinsing with PBS, the sections were dehydrated through a graded ethanol series. Finally, IHC staining results were visualized directly by DP80 CCD brightfield microscopy (Olympus, Japan). The outlines of the SNc, VTA, dorsal striatum (CPu), and nucleus accumbens (NAc) were delimited according to anatomical landmarks [26].

For immunofluorescence staining (IFC), sections were incubated for 1 h in blocking solution (5% normal goat serum, 0.2% Triton-X 100, and 0.05% NaN3 in PBS). The primary antibodies were used as follows: anti-Nurr1 (1:100, M196; Santa Cruz Biotechnology, USA), anti-TH (1:1000, AB152; Millipore, USA), anti-DAT (1:2000, MAB369; Millipore, USA), anti-TH (1:2000, T1299; Sigma-Aldrich, USA), anti-TH (1:1000, TYH, Aves Labs, USA), anti-GDNF (1:100, SC-13147; Santa Cruz Biotechnology, USA), anti-BDNF (1:100, SC-65514; Santa Cruz Biotechnology, USA), anti-Aldh1a1 (1:1000, AF1351; Beyotime Biotechnology, China), anti-NeuN (1:1000, MAB377; Millipore, USA), anti-cleaved caspase-3 (1:1000, 9664; CST, USA), anti-Iba1 (1:1000, 019-19741, FUJIFILM Wako Pure Chemical Corporation, Japan), anti-GFAP (1:100, sc33673; Santa Cruz Biotechnology, USA), and anti-Pitx3 (provided by Dr. Marten P. Smidt’s lab at the University of Amsterdam, Netherlands). For the immunostaining of Nurr1, GDNF, and BDNF, the blocking steps were performed after antigen retrieval (citrate buffer was made with 3 g trisodium citrate and 0.4 g citrate diluted in 1 L distilled water, pH 6.0). Finally, section images were visualized and photographed directly with a confocal microscope (A1 confocal, Nikon Instruments [Shanghai]Co., Ltd.) and a DP80 CCD brightfield microscope (Olympus, Japan). The outlines of the SNc and VTA were delimited according to anatomical landmarks [27].

### Image analysis

TH^+^ cells in the SNc and VTA were calculated in every three sections from −2.70 to −3.88 mm Bregma at 10× magnification by an observer who was blind to the genotype, and data were collected from 8 to 10 slices per animal. The IFC intensity and fiber density were analyzed using ImageJ software, and the data were collected from 2 to 3 slices per animal.

### High-performance liquid chromatography

Mouse brains were rapidly dissected, and the whole striatum in the left brain was isolated. For high-performance liquid chromatography (HPLC) analysis (EICOM, HTEC-500, USA), the tissue specimen was first weighed and sonicated on ice. DA was then extracted from the tissue homogenate and measured by reverse-phase HPLC through the electrochemical detection method. Different DA standard concentrations were detected to plot a standard curve for data analysis, as described previously [28].

### Statistical analysis

Data were expressed as means ± SEM and were analyzed using GraphPad Prism software (Version 7.0). Statistical comparisons were performed using non-parametric two-tailed Mann–Whitney test or Student’s two-tailed unpaired *t*-test as indicated in the figure legends, and *p* < 0.05 was considered significant. All experiments were repeated at least three times. No statistical methods were used to predetermine sample size, but our sample sizes are similar to those reported in previous publications.

## Results

### Selective deletion of Pitx3 in mature mdDA neurons

**Fig. 1:**
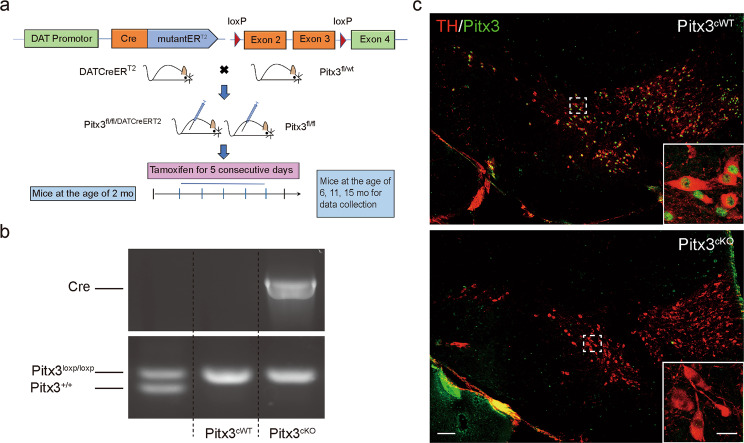
Conditional knockout of *Pitx3* in mdDA neurons. **a** The basic strategy for the generation of TAM-inducible *Cre*/*loxp*-directed *Pitx3* knockout mice. An experimental timeline for the administration of TAM and tissue collection is also presented. **b** PCR detection of *Cre* transgene (upper) and *Pitx3* floxed allele (lower). **c** IFC analysis for Pitx3 expression in mdDA neurons was performed using an antibody against Pitx3 (green) together with TH (red) in 4-month-old *Pitx3*^*cWT*^ and *Pitx3*^*cKO*^ mice (scale bar: 100 μm; high-magnification, 25 μm).

To investigate the role of Pitx3 in mature mdDA neurons, we first established a mouse model with selective *Pitx3* deletion in mdDA neurons using a TAM-inducible *Cre*^*ERT2/loxp*^ gene-targeting system (Fig. 1a). Cre-mediated recombination resulted in the removal of the second and third coding exons of *Pitx3* (Fig. 1a). After breeding, homozygous *Pitx3*-floxed mice harboring either no copies (*Pitx3*^cW*T*^) or a single copy of the *Cre*^*ERT2/loxp*^ gene (*Pitx3*^*cKO*^) were generated and characterized by conventional PCR analysis (Fig. 1b). At 2 months old, *Pitx3*^*cWT*^ and *Pitx3*^*cKO*^ mice were both injected intraperitoneally with TAM that provided a tightly spatial and temporal control of *Pitx3* deletion. Tissues were first collected at 2 months after TAM administration (4 months of age), and the Pitx3 expression profile in mdDA neurons was detected by IFC staining (Fig. 1c). As expected, the expression levels of Pitx3 in the mdDA neurons of 4-month-old *Pitx3*^*cKO*^ mice were not detectable, indicating the success of *Pitx3* deletion. In all subsequent experiments, *Pitx3*^*cKO*^ mice were treated with TAM at 2 months after birth, and the data were analyzed from three indicated time points (6, 11, and 15 months of age).

### Progressive mdDA neuronal loss and profound fiber pathology in *Pitx3*^*cKO*^ mice

**Fig. 2:**
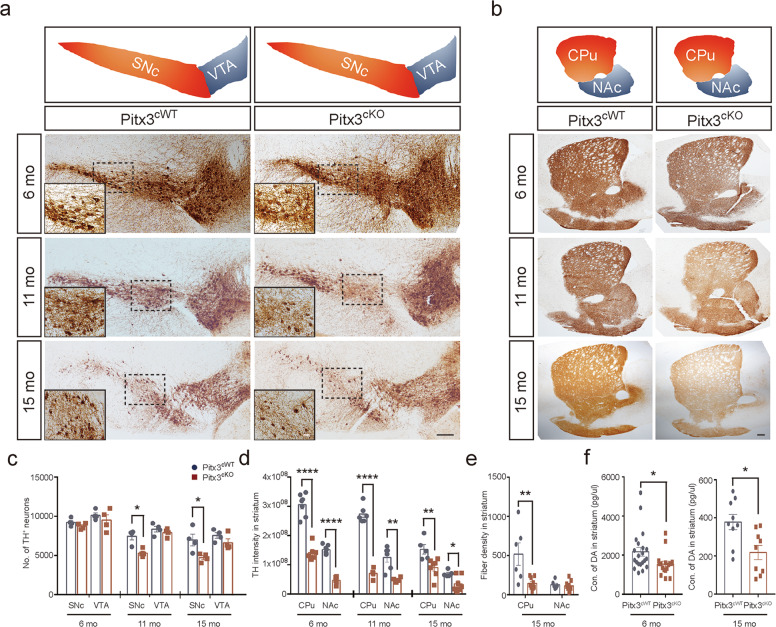
Neuropathological examination of the SNc-striatal pathway of *Pitx3*^*cKO*^ mice. **a** IHC staining of TH in the ventral midbrain sections from 6-, 11-, and 15-month-old *Pitx3*^*cWT*^ and *Pitx3*^*cKO*^ mice. SNc and VTA are highlighted with red and blue colors, respectively (scale bar: 100 μm; high-magnification, 50 μm). **b** IHC staining of TH in the striatal sections from 6-, 11-, and 15-month-old *Pitx3*^*cWT*^ and *Pitx3*^*cKO*^ mice. CPu and NAc are highlighted with red and blue colors, respectively (scale bar, 100 μm). **c** Quantification of TH^+^ neurons in the SNc and VTA from 6-, 11-, and 15-months-old *Pitx3*^*cWT*^ and *Pitx3*^*cKO*^ mice (*N* = 3-4 mice per genotype). Mann–Whitney test, *p = 0.0286 (11 months old for SNc); unpaired *t*-test, *p = 0.0299 (15 months old for SNc). **d** Quantification of TH IHC intensity in the striatum from 6-, 11-, and 15-months-old *Pitx3*^*cWT*^ and *Pitx3*^*cKO*^ mice (*N* = 4–7 mice per genotype). Unpaired *t*-test, ****p < 0.0001 (6 months old for CPu and NAc); ****p < 0.0001 (11 months for CPu); **p = 0.0053 (11 months for NAc); **p = 0.0095 (15 months for CPu); and *p = 0.0233 (15 months for NAc). **e** Quantification of TH^+^ fiber density in the striatum from 15-month-old *Pitx3*^*cWT*^ and *Pitx3*^*cKO*^ mice (*N* = 6–9 mice per genotype). Unpaired *t*-test, **p = 0.0078 (CPu). **f** Striatal DA levels in 6- and 15-month-old *Pitx3*^*cWT*^ and *Pitx3*^*cKO*^ mice (*N* = 9–21 mice per genotype). Mann–Whitney test, *p = 0.0148 (6 months old); and *p = 0.0102 (15 months old).

To further analyze the consequences of *Pitx3* deletion in mature mdDA neurons, we examined the number of mdDA neurons in the SNc and VTA of *Pitx3*^*cKO*^ mice at 6, 11, and 15 months of age following TAM administration. Notably, about 29% of SNc mdDA neurons were lost in 11-month-old *Pitx3*^*cKO*^ mice, and the deficit was exaggerated at 15 months of age with around 32% neuronal loss in *Pitx3*^*cKO*^ mice (Fig. 2a, c). However, VTA mdDA neurons were less affected by *Pitx3*-deficiency and remained intact during aging (Fig. 2a, c), resembling the neuropathological phenotype of *ak* mice [21]. Thus, our data re-emphasized that mdDA neurons consist of different subsets, each with a distinct vulnerability to neurodegeneration [4]. In addition to the neuron loss, *Pitx3*-deficiency also led to profound nerve fiber pathology. Our longitudinal data demonstrate that the striatal TH signals were progressively diminished in both *Pitx3*^*cWT*^ and *Pitx3*^*cKO*^ mice from 6 to 15 months of age (Fig. 2b, d). However, the lack of Pitx3 abnormally accelerated the decline at the early stages, i.e., IHC intensity of striatal TH was first noted to be dramatically decreased in 6-month-old *Pitx3*^*cKO*^ mice, whereas no further marked reduction was identified from 11 to 15 months of age (Fig. 2b, d). On the other hand, TH^+^ fiber density showed a significant loss in the dorsal striatum of 15-month-old *Pitx3*^*cKO*^ mice (Fig. 2b, e). The striatal DA levels were also dramatically reduced in *Pitx3*^*cKO*^ mice, approximately a 50% reduction compared with *Pitx3*^*cWT*^ mice at 15 months of age (Fig. 2f), suggesting that the disrupted dopaminergic innervation was closely associated with the alteration of DA content. Interestingly, as early as 6 months of age, the mean DA concentration was already reduced from 2174 pg/μL in *Pitx3*^*cWT*^ to 1545 pg/μL in *Pitx3*^*cKO*^ (Fig. 2f). However, the number of mdDA neurons was comparable between the two genotypes at this stage, suggesting that the perturbation of dopamine homeostasis may initially occur in axon terminals rather than in the soma.

**Fig. 3:**
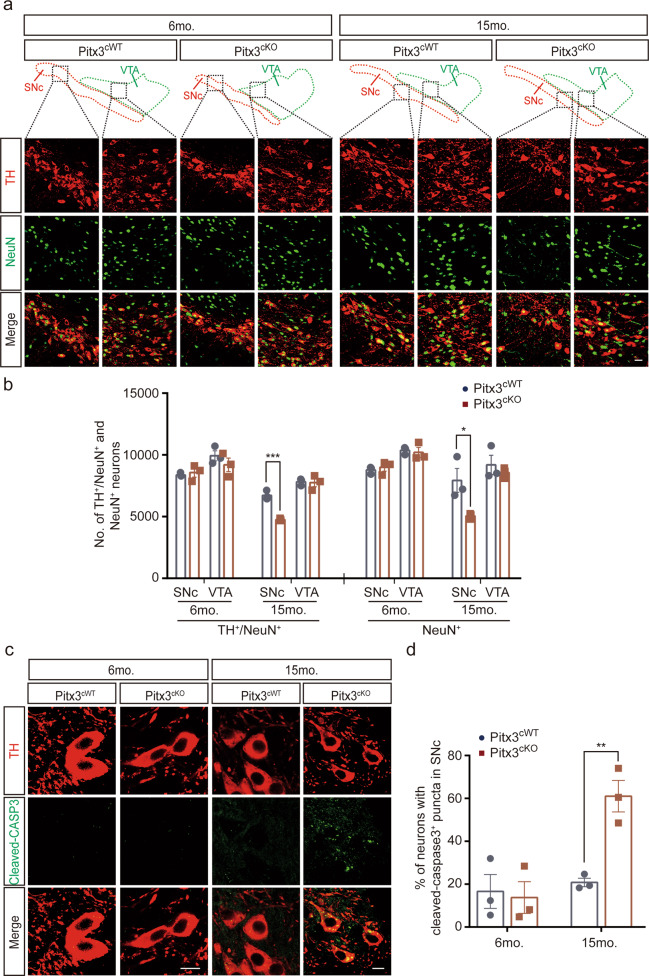
Neurodegeneration in 15-month-old *Pitx3*^*cKO*^ mice accompanied by the promoted apoptosis. **a** IFC co-staining of TH and NeuN, and IFC staining of NeuN in the ventral midbrain sections from 6 and 15-month-old *Pitx3*^*cWT*^ and *Pitx3*^*cKO*^ mice. SNc and VTA were outlined, respectively (scale bar: 10 μm). **b** Quantification of TH^+^/NeuN^+^ and NeuN+ neurons in the SNc and VTA from 6- and 15-months-old *Pitx3*^*cWT*^ and *Pitx3*^*cKO*^ mice (*N* = 3 mice per genotype). unpaired *t*-test, ***p = 0.0008 (15 months old for TH^+^/NeuN^+^ co-staining); unpaired *t*-test, *p = 0.0424 (15 months old for NeuN^+^ staining). **c** IFC staining of cleaved-caspase3 in the SNc area from 6 and 15-month-old *Pitx3*^*cWT*^ and *Pitx3*^*cKO*^ mice (scale bar: 10 μm). **d** Quantification of the percentage of neurons with cleaved-caspase3^+^ puncta in SNc (*N* = 3 mice per genotype). unpaired *t*-test, **p = 0.0062.

Since the downregulation of TH expressions was reported in *ak* mice previously [18], to make the identification of mdDA neurons more accurate, we also included another neuronal marker NeuN for neuron counting in the same SNc and VTA areas used for counting TH^+^ neurons [29]. Our results indicated that the number of NeuN^+^ cells was significantly lost, about 36% in the SNc area of 15-month-old *Pitx3*^*cKO*^ mice (Fig. 3a, b), whereas the number of SNc NeuN^+^ neurons was comparable between two genotypes at 6 months of age (Fig. 3a, b), mirroring the DAB staining results of TH in the SNc area and further proving that the neuronal death indeed occurs at the advanced stage. In contrast to SNc, the NeuN^+^ cells in the VTA of 15-month-old *Pitx3*^*cKO*^ mice were largely retained, and the two genotypes have comparable numbers of VTA NeuN^+^ cells (Fig. 3a, b). In addition, we examined the activation of caspase-3 in the SNc region, showing that more mdDA neurons in 15-month-old *Pitx3*^*cKO*^ mice displayed concentrated cleaved-caspase3 puncta, compared to the age-matched *Pitx3*^*cWT*^ mice (Fig. 3c, d), indicating that apoptosis may be promoted to trigger the neuronal death upon *Pitx3*-deficiency.

### Behavioral impairments in *Pitx3*^*cKO*^ mice

**Fig. 4:**
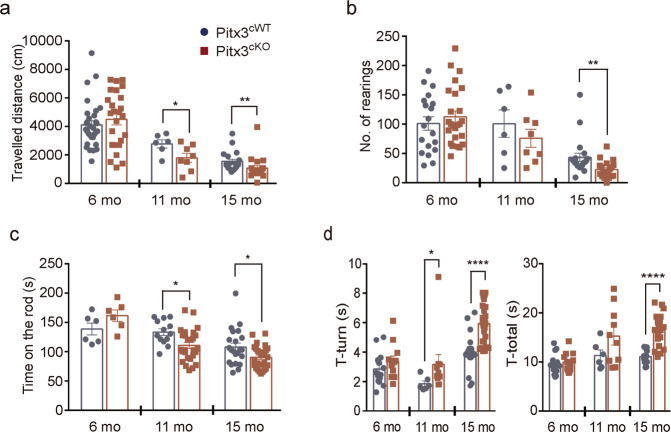
Motor behavior in *Pitx3*^*cKO*^ mice. **a** Total traveled distance and **b** the number of rearings were performed in open-field tests (*N* = 6–31 mice per genotype). Unpaired *t*-test, *p = 0.0471 (total distance at 11 months of the age); Mann–Whitney test, **p = 0.0011 (total distance at 15 months old); and **p = 0.0011 (rearings at 15 months old). **c** The latency to fall from rotarod for 6-, 11-, and 15-month-old *Pitx3*^*cWT*^ and *Pitx3*^*cKO*^ mice (*N* = 6–27 mice per genotype). Unpaired *t*-test, *p = 0.0107 (11 months old); and *p = 0.0206 (15 months old). **d** The turning time and total task time for 6-, 11-, and 15-month-old *Pitx3*^*cWT*^ and *Pitx3*^*cKO*^ mice in pole tests (*N* = 6–27 mice per genotype). Unpaired *t*-test, ****p < 0.0001 (turning time at 15 months old); and ****p < 0.0001 (total time at 15 months old); Mann–Whitney test, *p = 0.016 (turning time at 11 months old).

Consistent with the forementioned neuropathological changes, motor behaviors were abnormally altered in *Pitx3*^*cKO*^ mice. The open-field test demonstrated that starting at 11 months of age, a significant difference in the distance traveled was identified between *Pitx3*^*cWT*^ and *Pitx3*^*cKO*^ mice (Fig. 4a). The deficit worsened in 15-month-old *Pitx3*^*cKO*^ mice, together with a reduced number of rearing, indicating a progressive decline in locomotor activity with the progress of aging (Fig. 4a, b). The rotarod test indicated that young *Pitx3*^*cKO*^ mice performed equally well with age-matched *Pitx3*^*cWT*^ mice, whereas *Pitx3*^*cKO*^ mice showed markedly less stay-time on the rotating rod at the advanced stages (Fig. 4c). Moreover, in the vertical pole test, a prolonged turning time was observed in 11- and 15-month-old *Pitx3*^*cKO*^ mice, whereas a prolonged total task time was identified in 15-month-old *Pitx3*^*cKO*^ mice (Fig. 4d). These observed motor activity abnormalities in *Pitx3*^*cKO*^ mice correlate with the progressively diminished striatal DA levels during aging.

### Distinct cellular deficiency within SNc and VTA mdDA neurons of *Pitx3*^*cKO*^ mice

**Fig. 5:**
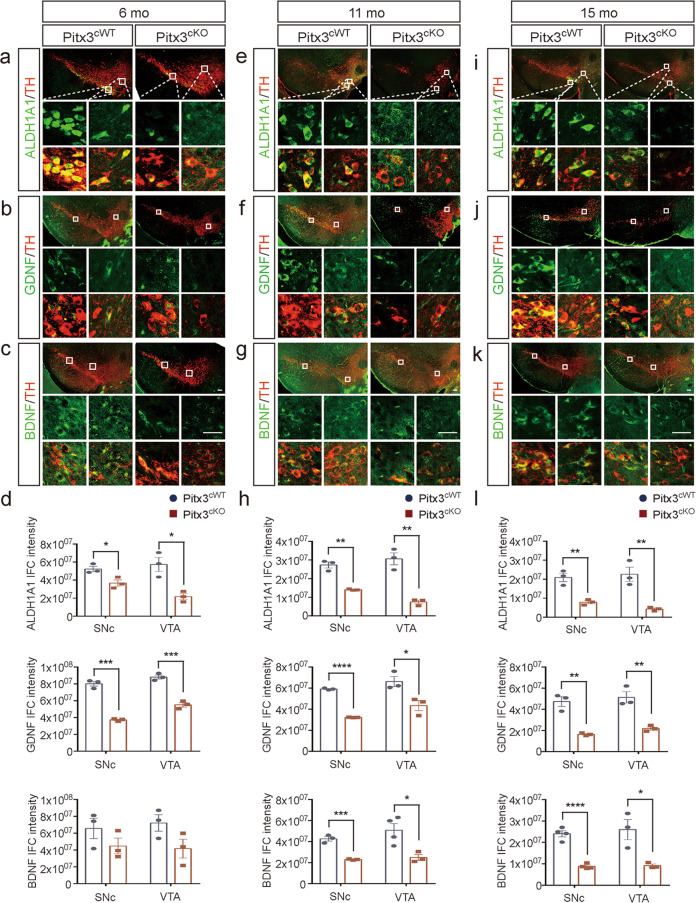
Expression analysis of *Pitx3*-related genes in *Pitx3*^*cKO*^ mice. Co-staining of Aldh1a1 and TH (**a**, **e**, **i**), GDNF and TH (**b**, **f**, **j**), and BDNF and TH (**c**, **g**, **k**) in the SNc and VTA from 6-, 11-, and 15-month-old *Pitx3*^*cWT*^ and *Pitx3*^*cKO*^ mice (scale bar: 100 μm; high-magnification, 25 μm). **d**, **h**, **l** Quantification of IFC intensity of Aldh1a1, GDNF, and BDNF in the SNc and VTA from 6-, 11-, and 15-month-old *Pitx3*^*cWT*^ and *Pitx3*^*cKO*^ mice (*N* = 3–4 mice per genotype). Unpaired *t*-test, *p = 0.0288 (Aldh1a1, 6 months old for SNc); ***p = 0.0001 (GDNF, 6 months old for SNc); *p = 0.0138 (Aldh1a1, 6 months old for VTA); ***p = 0.0007 (GDNF, 6 months old for VTA); **p = 0.0012 (Aldh1a1, 11 months old for SNc); ****p < 0.0001 (GDNF, 11 months old for SNc); ***p = 0.0002 (BDNF, 11 months old for SNc); **p = 0.0023 (Aldh1a1, 11 months old for VTA); *p = 0.0285 (GDNF, 11 months old for VTA); *p = 0.0225 (BDNF, 11 months old VTA); **p = 0.0052 (Aldh1a1, 15 months old for SNc); **p = 0.0026 (GDNF, 15 months old for SNc); ****p < 0.0001 (BDNF, 15 months old for SNc); **p = 0.0086 (Aldh1a1, 15 months old for VTA); **p = 0.0062 (GDNF, 15 months old for VTA); and *p = 0.0237 (BDNF, 15 months old for VTA).

**Fig. 6:**
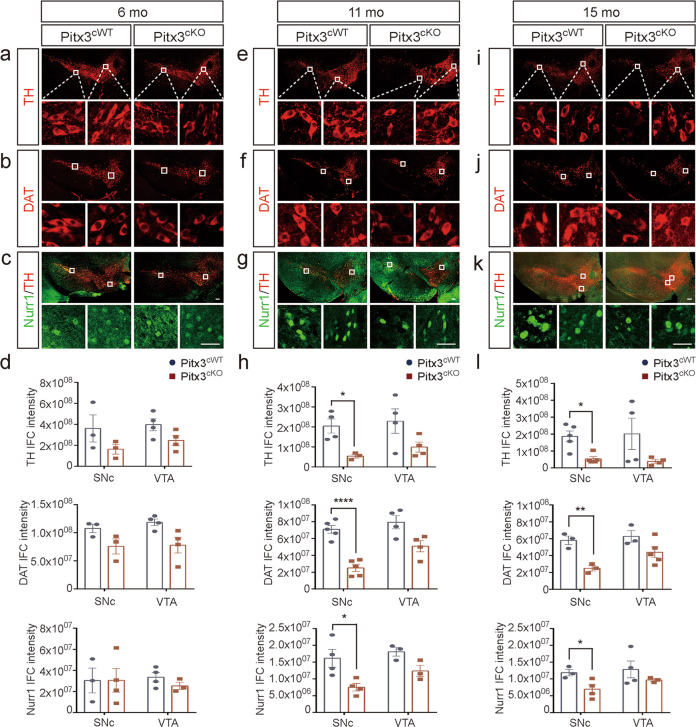
Expression analysis of mdDA neuronal markers in *Pitx3*^*cKO*^ mice. The IFC staining of TH (**a**, **e**, **i**), DAT (**b**, **f**, **j**) and the IFC co-staining of Nurr1 and TH (**c**, **g**, **k**) were presented in the SNc and VTA from 6-, 11-, and 15-month-old *Pitx3*^*cWT*^ and *Pitx3*^*cKO*^ mice (scale bar: 100 μm; high-magnification, 25 μm). **d**, **h**, **l** Quantification of TH, DAT, and Nurr1 IFC intensity in the SNc and VTA from 6-, 11-, and 15-month-old *Pitx3*^*cWT*^ and *Pitx3*^*cKO*^ mice (*N* = 3–5 mice per genotype). Unpaired *t*-test, *p = 0.0164 (TH, 11 months for SNc); ****p < 0.0001 (DAT, 11 months for SNc); *p = 0.0248 (Nurr1, 11 months for SNc); **p = 0.0051 (DAT, 15 months for SNc); and *p = 0.0395 (Nurr1, 15 months for SNc); Mann–Whitney test, *p = 0.0159 (TH, 15 months for SNc).

To further investigate cellular deficiency in different subtypes of mdDA neurons, a number of candidate genes were analyzed within SNc and VTA by immunostaining (Figs. 5 and 6). First, starting at 6 months of age Aldh1a1 and GDNF expressions were noted to decrease in both SNc and VTA mdDA neurons of *Pitx3*^*cKO*^ mice (Fig. 5a, b) and showed a steady reduction in the later stages (Fig. 5e, f, i and j). We speculated that such a decrease may be possibly due to the disruption of their regulatory pathway due to *Pitx3*-deficiency. BDNF was significantly reduced in both SNc and VTA mdDA neurons of 11- and 15-month-old *Pitx3*^*cKO*^ mice, but not at early stages (Fig. 5c, g and k), indicating that *Aldh1a1* and *GDNF* may be more dependent on *Pitx3* regulation than *BDNF*. On the other hand, a dramatic reduction in TH, DAT, and Nurr1 was observed in the SNc mdDA neurons of 11- and 15-month-old *Pitx3*^*cKO*^ mice, which could be resulted from the profound neuronal loss (Fig. 6). In contrast to their expression in SNc, TH, DAT, and Nurr1 levels were not significantly altered in the spared VTA mdDA neurons between *Pitx3*^*cKO*^ and *Pitx3*^*cWT*^ mice at all stages (Fig. 6), indicating that these genes may be less regulated by *Pitx3* in the adult VTA neurons. Altogether, the data suggest that distinct Pitx3-dependent pathways exist within SNc and VTA mdDA neurons, reflecting that *Pitx3*-deficiency specifically increases the vulnerability of SNc mdDA neurons, but not VTA, during adulthood. To better understand the distinct expression profiling of candidate genes within the mdDA neuronal subtypes, we summarized the above data in Supplementary Fig. 1.

### Aged *Pitx3*^*cKO*^ mice developed progressive neuropathological abnormalities on α-syn, microgliosis, and astrocytosis

**Fig. 7:**
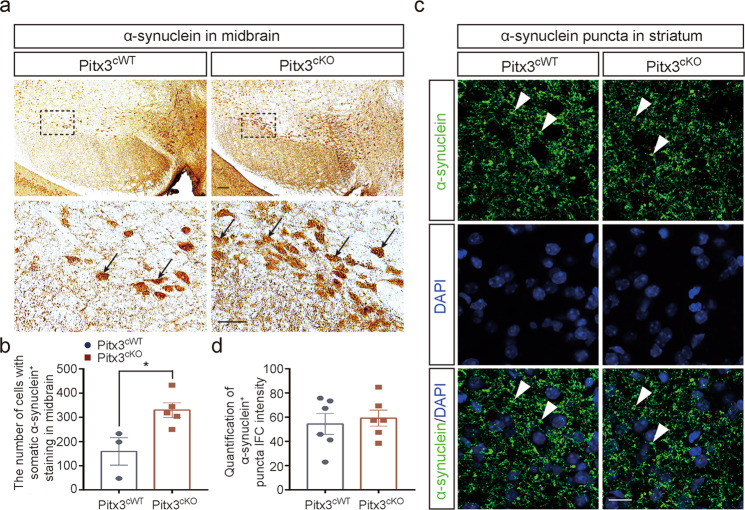
The increasing number of neurons with somatic accumulation of α-syn in 15-month-old *Pitx3*^*cKO*^ mice. **a** IHC staining of α-syn in the ventral midbrain from 15-month-old *Pitx3*^*cWT*^ and *Pitx3*^*cKO*^ mice (scale bar: 100 μm; high-magnification, 50 μm). **b** Quantification of α-syn^+^ cells in the ventral midbrain from 15-month-old *Pitx3*^*cWT*^ and *Pitx3*^*cKO*^ mice (*N* = 3–4 mice per genotype). Mann–Whitney test, *p = 0.0357. **c** IFC staining of α-syn in the striatum from 15-month-old *Pitx3*^*cWT*^ and *Pitx3*^*cKO*^ mice (scale bar: 10 μm). **d** Quantification of α-syn^+^ puncta IFC intensity in the striatum from 15-month-old *Pitx3*^*cWT*^ and *Pitx3*^*cKO*^ mice (*N* = 6 mice per genotype).

To further clarify the potential role of Pitx3 in PD-related pathology, we first examined the accumulation of endogenous α-syn by immunostaining. Increasing number of neurons with the somatic accumulation of α-syn were detected in 15-month-old *Pitx3*^*cKO*^ mice (Fig. 7a, b). α-syn^+^ neurons within SNc and VTA were previously reported in wild-type mice [30, 31]. Such accumulation was exacerbated in our 15-month-old *Pitx3*^*cKO*^ mouse model. Meanwhile, the data were closely correlated with the progression of neurodegeneration in *Pitx3*^*cKO*^ mice at this advanced stage, suggesting that the increased somatic accumulation of α-syn may trigger the pathogenic cascades leading to cell death. By contrast, no significant difference of α-syn staining was observed in the striatum of 15-month-old *Pitx3*^*cWT*^ and *Pitx3*^*cKO*^ mice (Fig. 7c, d). In addition, no apparent α-syn staining was detected in soma of striatal neurons of 15-month-old *Pitx3*^*cWT*^ and *Pitx3*^*cKO*^ mice.

Furthermore, we examined the brain sections of control and mutant animals for associated microgliosis and astrocytosis. The morphology of microglia was visualized by staining for ionized calcium-binding adapter molecule-1 (Iba1) [32]. The presence of reactive astrocytes was detected by staining for glial fibrillary acidic protein (GFAP) [33]. At 6 months of age, the reactive Iba1-positive cells were detected in both *Pitx3*^*cWT*^ and *Pitx3*^*cKO*^ mice, while more identified in *Pitx3*^*cKO*^ mice, which reached statistical significance compared with *Pitx3*^*cWT*^ mice. As expected, such phenotype aggravated in 15-month-old *Pitx3*^*cKO*^ mice. The ratio of reactive Iba1-positive cells in *Pitx3*^*cKO*^ mice increased to 30%, while around 8% in *Pitx3*^*cWT*^ (Supplementary Fig. 2a, b). Interestingly, a similar augment of reactive GFAP-positive cells was observed at the age of 15 months. The ratio of reactive GFAP-positive cells in *Pitx3*^*cKO*^ mice reached 48%, while around 30% in *Pitx3*^*cWT*^ (Supplementary Fig. 2a, c). However, no apparent increase of reactive astrocytes was observed in 6-month-old *Pitx3*^*cKO*^ mice compared to *Pitx3*^*cWT*^ mice. Taken together, these findings demonstrate that a significant exacerbation of astrocytosis and microgliosis may contribute to neurodegeneration observed in the SNc mdDA neurons of 15-month-old *Pitx3*^*cKO*^ mice.

## Discussion

The mdDA neurons modulate many brain functions, including movement, emotion, and reward [7, 34]. Their degeneration and dysfunction contribute to several neurological disorders, including PD [35]. Extensive studies have been conducted to determine the regulatory pathways involved in cell fate specification and differentiation of mdDA neurons [36, 37], and several transcription factors have been well described. Remarkably, these molecules continue to be expressed in mdDA neurons throughout life, though at lower levels than during development [38]. In contrast to their comprehensive documentation at early stages, little information is available on how these transcription factors affect mdDA neurons at late stages. Pitx3, a transcription factor essential for the postmitotic development of mdDA neurons, has been identified to be severely reduced within PD brain tissues as well as in PD patients’ peripheral blood lymphocytes [8, 39]. Furthermore, several *Pitx3* gene variants have been associated with sporadic PD [16, 40]. Thus, Pitx3 is critical not only for early differentiation but also for the maintenance of adult mdDA neurons. As an initial attempt to understand how Pitx3 contributes to the function of mature mdDA neurons, we established the *Pitx3*^*cKO*^ mouse model and reported for the first time that conditional knockout of *Pitx3* in fully differentiated mdDA neurons caused a rapid reduction of striatal DA, behavioral abnormalities, and progressive neuronal loss. Importantly, in our model, the striatal DA decline occurs long before mdDA neurons die, along with the distinct fiber pathology. These data suggest that perturbation on axonal terminals may be an early event in disease progression [41]. Taken together, the abnormalities caused by *Pitx3*-deficiency recapitulate the significant features of PD, suggesting that our mouse model can serve as a relevant PD model.

Our present studies provide a distinct, Pitx3-dependent expression profile in two subpopulations of mdDA neurons, namely SNc and VTA mdDA neurons, during aging. The data demonstrated that Pitx3 not only defines the molecular specification of neurons during early development but also maintains this characterization throughout adulthood. Pitx3 as a transcription factor is considered to modulate TH expression by binding to the 50 bp upstream area of the transcriptional start site [42]. During mdDA neuronal development, TH expression is initiated at the E11.5 stage, when *ak* mice show the comparable mdDA neuronal numbers with wild-type mice [21]. However, at E12.5 stage, the lateral TH^+^ neurons are largely lost in *ak* mice, and the TH^+^ signals are only detectable in a more dorsal field [21]. These studies indicate that Pitx3 is essential for the onset of TH expression in mdDA neurons during development [43]. However, what is the role of Pitx3 in TH regulation within adult mdDA neurons? In our *Pitx3*^*cKO*^ mouse model, TH expression markedly diminished in SNc mdDA neurons at 9 months after TAM treatment but remained unaltered in the spared VTA mdDA neurons. Meanwhile, a moderate loss of mdDA neurons was also first noted at this stage. We thereby speculated that the severe neuronal loss within SNc may contribute to significant TH reduction. On the other hand, the unaltered TH levels within the VTA mdDA neurons of *Pitx3*^*cKO*^ mice may be due to continuous Nurr1 expression. Nurr1, another regulator of TH [44], remained steadily expressed in the VTA mdDA neurons of 11- and 15-month-old *Pitx3*^*cKO*^ mice, which may compensate for the on-site Pitx3 ablation and contribute to the unaltered TH expression within the VTA mdDA neurons at the advanced stages. Taken together, our study findings demonstrate that in adult mdDA neurons, *Pitx3*-deficiency may cause progressive TH reduction and significant neuronal loss in SNc. Conversely, in the spared VTA mdDA neurons, TH is steadily expressed even at the advanced stages, indicating that an alternative regulatory pathway in VTA may exist, possibly contributed by uninterrupted Nurr1 expression. We speculated that Nurr1 may play a vicarial role in justifying the late events during aging. Our data further indicate that the distinct vulnerability of mdDA neuronal subtypes is delicately controlled by the Pitx3-associated regulatory pathways during adulthood.

A follow-up question is why the mdDA neurons show progressive degeneration upon *Pitx3*-deficiency. Dramatically concentrated cleaved-caspase3 was characterized in *Pitx3*^*cKO*^ mice, indicating that *Pitx3*-deficiency might promote the apoptosis of mdDA neurons. On the other hand, we have previously described that GDNF, BDNF, and Pitx3 are engaged in a feedforward regulatory pathway during development [45]. In our present study, the remarkable reduction of GDNF in SNc mdDA neurons appeared as early as 6 months in *Pitx3*^*cKO*^ mice and continued to decrease into the advanced stages. As reported, GDNF is required for mdDA neuronal survival during development [46], we thereby speculate that such early GDNF deficit may contribute to late neuronal degeneration. Conversely, BDNF was steadily expressed in SNc mdDA neurons in both *Pitx3*^*cWT*^ and *Pitx3*^*cKO*^ mice at the early stages, but eventually decreased at the advanced stages, suggesting that a weaker association may exist between BDNF and Pitx3 during adulthood. Our model thus indicates that the feedforward interaction with GDNF, BDNF, and Pitx3 may not only protect mdDA neurons during embryogenesis but also promote neuronal survival during adulthood. Similarly, Aldh1a1, as a downstream target of Pitx3, is also reduced in 6-month-old *Pitx3*^*cKO*^ mice. *Aldh1a1* encodes for the retinoic acid-synthesizing enzyme and oxidizes the highly reactive DA catabolic intermediate dopamine, 3,4-dihydroxyphenylacetaldehyde (DOPAL), to prevent cytotoxicity [47]. Therefore, the decrease in Aldh1a1 may disrupt the DA metabolic equilibrium, resulting in mdDA neuronal dysfunction at the early stages. In addition, *Aldh1a1*-deficiency was reported to trigger α-syn aggregation in A53T transgenic mice [48, 49]. In our mouse model, the increasing number of neurons with the somatic accumulation of α-syn were characterized in 15-month-old *Pitx3*^*cKO*^ mice, which was not previously recognized in the conventional *Pitx3* knockout mouse lines. We thereby speculated that a severe loss of Aldh1a1 expression caused by *Pitx3*-deficiency may highly likely result in DOPAL formation, further increasing the somatic accumulation of α-syn [50, 51]. These data suggest that the reduction in GDNF and Aldh1a1 levels may cause neuronal dysfunction and disrupt DA metabolism within SNc mdDA neurons at the early stages of *Pitx3*^*cKO*^ mice, possibly contributing to late neurodegeneration. On the other hand, the increased somatic accumulation of α-syn and reduced BDNF levels may promote the apoptosis and exacerbate the PD-related pathologies in *Pitx3*^*cKO*^ mice at the advanced stages.

Taken together, the conditional knockout of *Pitx3* in fully differentiated adult mdDA neurons results in progressive neurodegeneration, motor abnormalities, and promoted apoptosis. We propose that the early reduction in GDNF and Aldh1a1 levels may be associated with late neuronal loss via the disruption of both neuronal function and DA metabolism. In addition, the increased somatic accumulation of α-syn may exacerbate PD-related pathology at the advanced stages. Furthermore, *Pitx3*-deficiency triggers the differential expression of candidate genes within the distinct subtypes of mdDA neurons, indicating that distinct neuronal identities are delicately regulated through Pitx3-dependent pathways during adulthood. However, more detailed mechanistic studies are required to further elucidate how neuronal specification maintains in the subtypes of adult mdDA neurons.

## Supplementary information

Supplementary Figure 1

Supplementary Figure 2

## Data Availability

All reagents will be available upon request.
