# Supplementary material for: The essential role of transcription factor Pitx3 in preventing mesodiencephalic dopaminergic neurodegeneration and maintaining neuronal subtype identities during aging
Source: Cell Death Dis. 2021 Oct 27;12(11):1008. doi: 10.1038/s41419-021-04319-x (PMC8551333; doi:10.1038/s41419-021-04319-x)
Supplement: Supplementary file 1 — Supplementary Figure 1 [file 41419_2021_4319_MOESM1_ESM.docx]

**Supplementary Figure 1.** Expression profiling of six candidate genes within SNc and VTA during aging, including TH, DAT, Nurr1, Aldh1a1, GDNF, and BDNF. The IFC intensity of six candidate genes was examined in SNc and VTA from 6-, 11-, and 15-month-old *Pitx3^cWT^* and *Pitx3^cKO^* mice. To simplify things, the distribution patterns of the different genes within SNc and VTA are roughly described in schematic images, but not their exact localization to distinct neuronal subtypes.

SNc mdDA neurons with a higher expression level of specific genes. SNc mdDA neurons with a lower expression level of specific genes. VTA mdDA neurons with a higher expression level of specific genes. VTA mdDA neurons with a lower expression level of specific genes. TH, tyrosine hydroxylase; DAT, dopamine transporter; Aldh1a1, aldehyde dehydrogenase 1 family member 1; GDNF, glial cell-derived neurotrophic factor; BDNF, brain-derived neurotrophic factor; IFC, immunofluorescence; SNc, substantia nigra pars compacta; VTA, ventral tegmental area; mdDA, mesodiencephalic dopaminergic.
