# Supplementary material for: The essential role of transcription factor Pitx3 in preventing mesodiencephalic dopaminergic neurodegeneration and maintaining neuronal subtype identities during aging
Source: Cell Death Dis. 2021 Oct 27;12(11):1008. doi: 10.1038/s41419-021-04319-x (PMC8551333; doi:10.1038/s41419-021-04319-x)
Supplement: Supplementary file 2 — Supplementary Figure 2 [file 41419_2021_4319_MOESM2_ESM.docx]

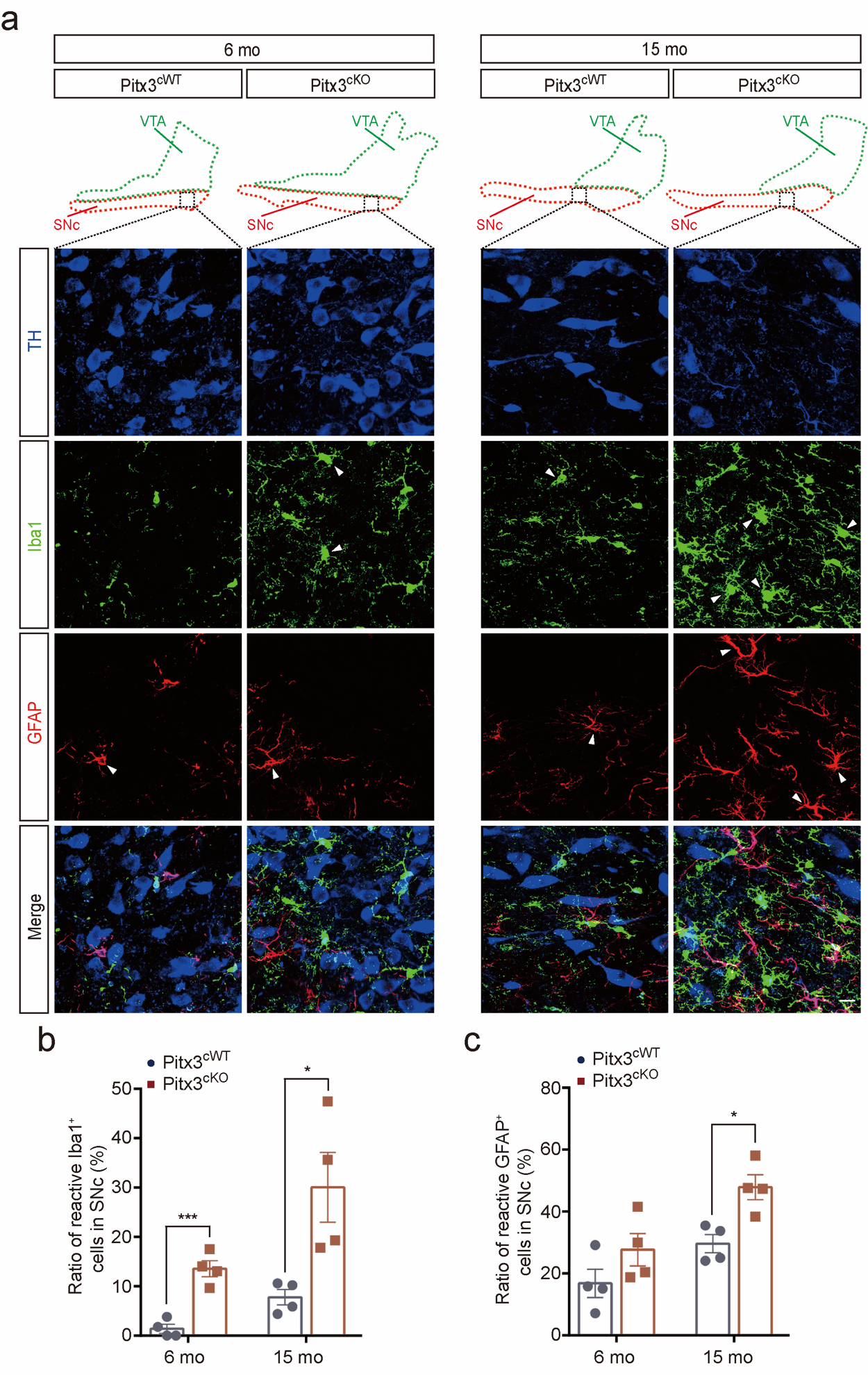


**Supplementary Figure 2.** Reactivity of microglia and astrocytes in the SNc of *Pitx3^cKO^* mice. **a** IFC staining of TH, Iba1 and GFAP in the SNc areas from 6- and 15-month-old *Pitx3^cWT^* and *Pitx3^cKO^* mice (Scale bar: 10 μm). The solid arrow points to a reactive cell. **b, c** Quantification of the ratio of reactive microglia **(b)** and astrocytes **(c)** in the SNc areas from 6- and 15-month-old *Pitx3^cWT^* and *Pitx3^cKO^* mice (N = 4 mice per genotype). Unpaired t-test, ****p* = 0.0006 (Iba1, 6 months old); **p* = 0.0217 (Iba1, 15 months old); **p* = 0.0106 (GFAP, 15 months old).
